# NMR resonance assignments for the active and inactive conformations of the small G protein RalA

**DOI:** 10.1007/s12104-019-09925-7

**Published:** 2020-01-08

**Authors:** Arooj Shafiq, Louise J. Campbell, Darerca Owen, Helen R. Mott

**Affiliations:** 1 Department of Biochemistry, 80, Tennis Court Road, Cambridge, CB2 1GA UK; 2 Present Address: Barrett Hodgson University, Korangi Creek, Salim Habib Campus, NC-24, Deh Dih, Korangi Creek, Karachi, 74900 Sindh Pakistan

**Keywords:** Ras, Gtpase, Ral, Signalling, G Protein

## Abstract

The Ral proteins (RalA and RalB) are small G proteins of the Ras family that have been implicated in exocytosis, endocytosis, transcriptional regulation and mitochondrial fission, as well as having a role in tumourigenesis. RalA and RalB are activated downstream of the master regulator, Ras, which causes the nucleotide exchange of GDP for GTP. Here we report the ^1^H, ^15^ N and ^13^C resonance assignments of RalA in its active form bound to the GTP analogue GMPPNP. We also report the backbone assignments of RalA in its inactive, GDP-bound form. The assignments give insight into the switch regions, which change conformation upon nucleotide exchange. These switch regions are invisible in the spectra of the active, GMPPNP bound form but the residues proximal to the switches can be monitored. RalA is also an important drug target due to its over activation in some cancers and these assignments will be extremely useful for NMR-based screening approaches.

## Biological context

The Ral small G proteins are members of the Ras superfamily and are regulated by Ras, which activates guanine nucleotide exchange factors (GEFs) that act on the Ral proteins. The RalGEFs sanction the removal of GDP from the Ral proteins, thereby permitting its replacement by GTP, which is more abundant in the cell (Bodemann and White 2008; Cooper et al. 2013). Once GTP-bound, the Ral proteins are active and can interact with various downstream effectors, including: components of the exocyst complex, Sec5 (Fukai et al. 2003) and Exo84 (Jin et al. 2005); RalBP1 (also known as RLIP76) (Fenwick et al. 2010); the Y-box transcription factor ZONAB (Frankel et al. 2005); and phospholipases C-δ1 (Sidhu et al. 2005) and D1 (Jiang et al. 1995). The Ral proteins regulate a variety of cellular processes through these effector molecules including exocytosis, endocytosis, actin cytoskeletal dynamics, transcriptional activation and mitochondrial fission. In addition, Ral proteins have been implicated in the initiation and regulation of oncogenic transformation of human cells (van Dam and Robinson 2006). The Ral proteins like other small G proteins act as molecular switches, which cycle between GTP bound active and GDP bound inactive states. They contain two dynamic switch regions [switch I (residues 41–51) and switch II (residues 69–81)], which are involved in effector binding and undergo conformational changes upon nucleotide exchange (Milburn et al. 1990). There are two Ral isoforms, RalA and RalB, which have approximately 85% sequence identity and in vitro bind with similar affinities to their effectors (Campbell et al. 2015). In vivo however, RalA and RalB appear to perform distinctive functions in normal cells and to play different roles in tumourigenesis and cancer progression. The sequence differences are mainly within the C-terminal, hypervariable region of RalA and RalB, and indeed all of the residues known to contact effector proteins are conserved (Mott and Owen 2010). There is also a single residue insertion in the loop between helix α3 and strand β5, which, while not contacting effectors directly, is close to the effector binding regions. These differences are likely to determine differential Ral protein interactions with their effector molecules (Campbell et al. 2015; Shipitsin and Feig 2004). Furthermore, the divergent C-terminal region of Ral proteins is also thought to determine their cellular localization (Shipitsin and Feig 2004).

The resonance assignments for RalB·GMPPNP were published in 2007 (Prasannan et al. 2007) but there are no resonance assignments currently available for RalA. Here, we report the backbone and sidechain assignments for RalA bound to the non-hydrolysable GTP analogue, GMPPNP. We also report the backbone amide assignments for the GDP bound, inactive form of RalA.

## Methods and experiments

Uniformly ^15^ N- and ^15^ N,^13^C-labelled simian RalA G domain (residues 1–184) containing the activating Q72L mutation was expressed and purified as described earlier (Campbell et al. 2015). The bound nucleotide was exchanged for GMPPNP (Sigma) as described previously (Thompson et al. 1998). Uniformly ^15^ N and ^15^ N-^13^C wild type (wt) RalA G domain (residues 1–184) was also expressed and purified as described above. To prepare a protein sample bound only to GDP, wt RalA was incubated at 25 °C for 16–20 h to allow complete hydrolysis of the bound GTP. HPLC analysis was used to confirm the identity of all bound nucleotides.

NMR samples contained 0.26 mM and 0.45 mM of ^15^ N- and ^15^ N,^13^C-labelled Q72L RalA·GMPPNP respectively or 0.75 mM of ^15^ N-labelled wt RalA·GDP in NMR buffer (20 mM Tris–HCl, pH 7.6, 150 mM NaCl, 1 mM MgCl_2_, 0.05% NaN_3_) with an additional 10% D_2_O. All NMR spectra were acquired on a Bruker DRX500 spectrometer at 298 K except ^13^C HSQC, HCCH-TOCSY and ^13^C-separated NOESY experiments, which were recorded on a Bruker DRX800 at 298 K. The following experiments were recorded on ^15^ N,^13^C-labelled Q72L RalA·GMPPNP: HNCA, HN(CO)CA, HNCACB, CBCA(CO)NH, HCCH-TOCSY, ^13^C HSQC, and ^13^C-separated NOESY. ^15^ N HSQC, ^15^ N-separated TOCSY and ^15^ N-separated NOESY experiments were recorded on ^15^ N-labelled samples of both Q72L RalA·GMPPNP and wt RalA·GDP. A 2D homonuclear ^1^H NOESY was recorded on 0.70 mM unlabelled Q72L RalA·GMPPNP. All NOESY experiments were recorded with 100 ms mixing times. NMR data were processed using AZARA (Boucher, https://www.bioc.cam.ac.uk/azara), and analysed using CCPN Analysis (Vranken et al. 2005).

For Q72L RalA·GMPPNP the backbone assignments were achieved using standard triple resonance methodology (Gardner and Kay 1998).The sidechain resonances were assigned using the triple resonance experiments (HNCACB, CBCA(CO)NH), in combination with ^15^ N-separated TOCSY and HCCH-TOCSY experiments, as well as ^15^ N-separated and ^13^C-separated NOESY. Sidechain resonances of aromatic residues were assigned using the aromatic region of a ^13^C HSQC and the relevant planes in the ^13^C-separated NOESY. Assignments were validated by the presence of return peaks in the related ^13^Cβ planes. The methyl groups of methionine were identified using their strong peaks at distinct chemical shifts in the ^13^C HSQC and the sign of the crosspeaks in constant time ^13^C HSQC. Sidechain amide resonances for Asn, Gln and Arg were assigned using the ^15^ N-separated and ^13^C-separated NOESY spectra. The 2D homonuclear ^1^H NOESY was used to identify proton chemical shifts for the bound GMPPNP. The backbone resonances for wt RalA·GDP were assigned with the aid of the assignments of Q72L RalA·GMPPNP, in combination with the ^15^ N-separated NOESY and TOCSY and ^15^ N HSQC recorded on both proteins (Clore and Gronenborn 1998a, b; Marion et al. 1989).

## Assignments and data deposition

Backbone resonances were never observed for the 11 N-terminal residues (1–11) of RalA. Subsequent electrospray ionisation mass spectrometry (ESI–MS) analysis showed that the N-terminus of RalA undergoes proteolysis in solution. This region is not the part of G-domain of Ral proteins (Bourne et al. 1991) and was similarly absent (and therefore unassigned) in RalB (Fenwick et al. 2009; Prasannan et al. 2007).

**Fig. 1:**
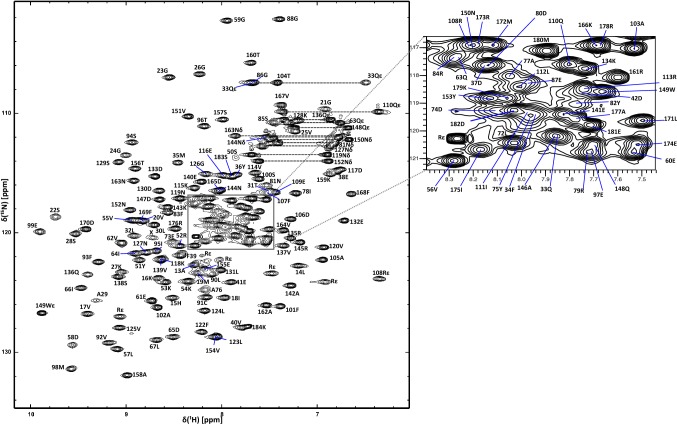
^1^H-^15^ N HSQC spectrum of RalA·GMPPNP at 298 K and 500 MHz

The complete backbone assignments were obtained for residues 12–184 of Q72L RalA·GMPPNP except for residues 43–49, which are within switch I, and 68–70, which are in switch II. The statistics for the backbone assignments are shown in Table 1. The ^1^H–^15^N HSQC spectrum showing the spectral quality and the assignments is shown in Fig. 1. The switch I (41–51) and switch II (68–81) regions in small G proteins exhibit internal mobility in the active form, on a timescale that makes their assignment problematic (Feltham et al. 1997; Kraulis et al. 1994; Mott et al. 1999). The missing assignments in these regions were therefore not surprising.

**Table 1:** Extent of assignments of RalA in the active (GMPPNP) and inactive (GDP) forms

	Atom	Number of expected	Number assigned	Percentage assignment (%)
RalA·GMPPNP (Q72L)	HN	171	162	94.7
	^15^NH	173	162	93.6
	^13^C	173	165	95.4
RalA·GDP	HN	171	169	98.8
	^15^NH	173	169	97.7

Sidechain assignments were achieved for all residues with observable backbone resonances in Q72L RalA·GMPPNP. In addition, sidechains were assigned for residues Thr69 Ala70 in switch II. Outside the two switch regions, aliphatic side-chains for residues 11 to 184 were almost fully assigned except for sidechain carbons of Lys27, Arg176 and Lys179. The ^1^H and ^13^C of all proton-bearing aromatic sidechains were fully assigned, except Tyr43 in switch I. Hε/Nε were assigned for Arg108 and Arg135, Hη was assigned for Tyr153 and OH were assigned for Thr31, Ser100, Ser129 and Thr160. All Gln and Asn sidechain NH_2_ groups were also assigned.

The H1, H2 and H8 resonances of the purine ring and H1′ of the ribose in GMPPNP were assigned using a 2D ^1^H NOESY spectrum. Assignments of RalA·GMPPNP have been deposited in the BMRB database, accession number 28046.

**Fig. 2:**
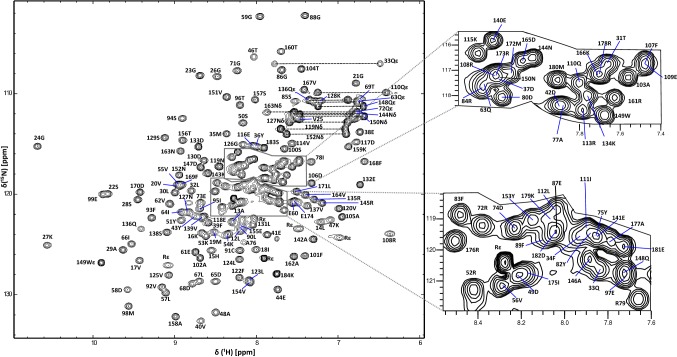
^1^H-^15^ N HSQC spectrum of RalA·GDP at 298 K and 500 MHz

For wt RalA·GDP, backbone amides of all of switch 1 could be assigned and assignments were missing only for Ala70 and Asn81 in switch II. The statistics for the backbone assignments are shown in Table 1. The ^1^H–^15^N HSQC spectrum of wt RalA·GDP showing the spectral quality and the assignments is shown in Fig. 2. Assignments of wt RalA·GDP have been deposited in the BMRB database, accession number 28047.
